# Gelatin-Based Soft-Tissue Sarcoma Organoids Recapitulate Patient Tumor Characteristics

**DOI:** 10.34133/bmr.0293

**Published:** 2025-12-09

**Authors:** Kyuyoung Han, Jiwan Choi, Chae-eun Kim, Seoon Kang, Hye-in An, Chan-Gi Pack, Jin-Hee Ahn, Hyunwook Kwon, Chan Wook Kim, Joon Seon Song, Tae Won Kim, Eunyoung Tak, Jeong Eun Kim

**Affiliations:** ^1^ Department of Oncology, Asan Medical Center, University of Ulsan College of Medicine, Seoul, Republic of Korea.; ^2^ Department of Convergence Medicine, Asan Medical Center, University of Ulsan College of Medicine, Seoul, Republic of Korea.; ^3^ Department of Medical Science, Asan Medical Institute of Convergence Science and Technology, Asan Medical Center, University of Ulsan College of Medicine, Seoul 05505, Republic of Korea.; ^4^ Department of Biomedical Engineering, University of Ulsan College of Medicine, Seoul 05505, Republic of Korea.; ^5^ Division of Kidney and Pancreas Transplantation, Department of Surgery, Asan Medical Center, University of Ulsan College of Medicine, Seoul, Republic of Korea.; ^6^ Division of Colon and Rectal Surgery, Department of Surgery, University of Ulsan College of Medicine, Asan Medical Center, Seoul, Republic of Korea.; ^7^ Department of Pathology, University of Ulsan College of Medicine, Asan Medical Center, Seoul, Republic of Korea.; ^8^ Asan Preclinical Evaluation Center for Cancer TherapeutiX, Asan Medical Center, Seoul 05505, Republic of Korea.

## Abstract

Soft-tissue sarcoma (STS) is a rare and heterogeneous group of cancers with more than 100 histological subtypes, which makes biological understanding and therapeutic development particularly challenging. Patient-derived tumor organoid models have transformed cancer research by providing patient-representative preclinical platforms, yet their application in STS has been limited because of low establishment efficiency. To address this problem, a gelatin-based culture protocol was developed to enhance critical cellular processes, including mitochondrial function and cell adhesion, which are essential for organoid self-organization. Using this optimized system, patient-derived tumor organoids were successfully established from representative STS subtypes, such as dedifferentiated liposarcoma and leiomyosarcoma. These organoids retained the histopathological architecture and molecular characteristics of the original tumors and reflected subtype-specific oncogenic pathways, mitochondrial dynamics, and lipid metabolic signatures. Our established gelatin-based organoid culture system enables efficient establishment of patient-derived organoids from representative STS subtypes, faithfully preserving their histopathological and molecular characteristics. These models recapitulate subtype-specific oncogenic pathways, mitochondrial dynamics, and lipid metabolic signatures, providing a robust and clinically relevant preclinical platform for investigating sarcoma biology and developing personalized therapeutic strategies.

## Introduction

Soft-tissue sarcoma (STS) is a rare and heterogeneous group of mesenchymal malignancies comprising more than 100 histological subtypes, each defined by distinct molecular and morphological features that complicate accurate diagnosis and treatment [1]. The current standard of care involves surgical resection, combined with radiotherapy, chemotherapy, or both [2]. However, the rarity, complexity, and subtype diversity of STS underscore the urgent need for preclinical, patient-specific models to improve therapeutic outcomes and elucidate underlying molecular mechanisms.

Organoids, 3-dimensional (3D) in vitro culture systems derived from self-organizing cells, have emerged as powerful tools for reproducing the structural and functional characteristics of native tissues [3]. Patient-derived tumor organoids (PDTOs) faithfully reflect the phenotypic and genetic features of primary tumors and have been successfully established for colorectal, breast, and lung cancers, proving instrumental for drug screening and personalized therapy development [4]. However, their application in STS research remains limited due to technical limitations.

Most PDTO generation typically relies on basement membrane extract (BME), a gelatinous protein mixture that mimics the extracellular matrix (ECM) and supports organoid growth [5]. However, BME has shown limited effectiveness in STS, prompting the search for alternative scaffolds [6].

Recently, gelatin-based systems have emerged as promising substitutes. As a collagen-derived biomaterial, gelatin offers excellent biocompatibility and ECM-like properties, promoting cell adhesion, proliferation, and survival in organoid cultures [7]. Given the mesenchymal origin of STS, gelatin provides a particularly appropriate scaffold. Widely used as a substrate for mesenchymal stem cell cultures, it facilitates cell aggregation, a critical step in 3D structure formation, while enhancing cell proliferation and mitochondrial function [8,9]. Based on these advantages, gelatin is hypothesized to be an optimal substrate for STS PDTO development. This approach successfully yielded robust and reproducible organoid models that closely recapitulated the histological and molecular features of primary STS tumors.

In this study, the establishment of STS PDTOs using a gelatin-based organoid culture system is reported. These organoids retained the histological and molecular characteristics of their parental tumors, supporting their utility as reliable preclinical platforms for investigating STS biology, therapeutic resistance, and patient-specific treatment strategies.

## Materials and Methods

### Patient sample collection

Tissue specimens of STS were obtained from 3 consenting patients who had undergone surgical resection at Asan Medical Center, Seoul, Republic of Korea. The research protocol was approved by the Ethics Committee of the Institutional Review Board (IRB) of Asan Medical Center (IRB approval number: AMCIRB. 2024-0156). Patient characteristics are summarized in Table S1, with detailed descriptions below.

#### Patient 1

A 40-year-old man was diagnosed with dedifferentiated liposarcoma (DDLPS) involving the sigmoid and distal colon. Initial resection was performed on 2021 February 26; however, the residual tumor remained. A second surgery (including tumor resection and left nephrectomy) was conducted on 2021 April 28; however, complete excision was not achieved. Palliative chemotherapy was initiated using the CYVAD (cyclophosphamide, vincristine, Adriamycin, dacarbazine) regimen, followed by second-line docetaxel and gemcitabine, which resulted in tumor shrinkage. On 2024 March 29, residual mass excision was performed, and tumor tissue was collected for organoid culture. Despite subsequent recurrence and additional surgery, pembrolizumab-based immunotherapy proved ineffective, and the patient was transferred to hospice care.

#### Patient 2

A 51-year-old male patient was diagnosed with retroperitoneal DDLPS and well-differentiated liposarcoma (WDLPS). Initial computed tomography revealed a large abdominal mass exceeding 30 cm, occupying most of the abdominal cavity. On 2024 August 9, surgical excision was performed, removing 2 distinct masses measuring 32 × 26 × 8 cm and 2.5 cm in diameter, respectively. Pathological evaluation confirmed DDLPS with positive resection margins. Postoperative management included 6 cycles of adjuvant chemotherapy using the Adriamycin–ifosfamide regimen, completed on 2024 December 24. The patient remains under regular follow-up.

#### Patient 3

An 81-year-old woman with retroperitoneal leiomyosarcoma (LMS) (FNCLCC grade 3) underwent an operation on 2024 April 22, which included mass excision, right nephrectomy, and inferior vena cava venoplasty. The tumor, measuring 6.5 × 6 × 4.8 cm, involved the renal vein and the inferior vena cava adventitia. Chemotherapy was not administered. Follow-up imaging through December 2024 showed no recurrence. Tumor tissue was collected for organoid generation.

### Liposarcoma cell line

The liposarcoma (LPS) cell line 93T449 (Research Resource Identifier: CVCL_U614) was obtained from the American Type Culture Collection (Manassas, VA, USA). Cells were cultured in Roswell Park Memorial Institute 1640 medium (Welgene Inc., Gyeongsan-si, Korea) supplemented with 10% fetal bovine serum (FBS; Thermo Fisher Scientific Inc., Waltham, MA, USA) and 1% penicillin–streptomycin (Cytiva, Marlborough, MA, USA). Before experiments, cells were screened for mycoplasma contamination. Subculturing was performed every 3 d at a split ratio of 1:3 to 1:4.

### Primary cell isolation and culture

Primary STS cells were isolated from freshly resected patient tissues. Immediately after surgical removal, specimens were transported to the Asan Pathology Department, where a pathologist confirmed the diagnosis within 1 h. The resected samples were then transported on ice to the laboratory, suspended in cold serum-free Dulbecco’s modified Eagle medium (DMEM)/F12 (Gibco) supplemented with antibiotics. Tumor tissues were then minced into small fragments and enzymatically digested using Enzymes H, R, and A from the Human Tumor Dissociation Kit (Miltenyi Biotec GmbH, Bergisch Gladbach, Germany). Digestion was performed in gentleMACS C Tubes (Miltenyi Biotec) using gentleMACS Dissociator (Miltenyi Biotec) for an hour. The resulting suspension was filtered through a 100-μm sterile cell strainer (SPL Life Sciences Co., Ltd., Pocheon, South Korea) and centrifuged at 500 × g for 5 min at room temperature. The cell pellet was washed 3 times in complete medium and then seeded at 70% to 80% confluency. Primary STS cells were cultured in DMEM/F12 medium, supplemented with 10% FBS (Thermo Fisher Scientific Inc.), 1% penicillin–streptomycin (Cytiva), 50 ng/ml human epidermal growth factor (Novoprotein Scientific Inc., Summit, NJ, USA), and 1× B27 supplement minus vitamin A (Gibco). Cells were manually passaged at 1:3 to 1:5 dilutions every 3 to 4 d.

### Establishment of sarcoma patient-derived organoids

**Fig. 1.**
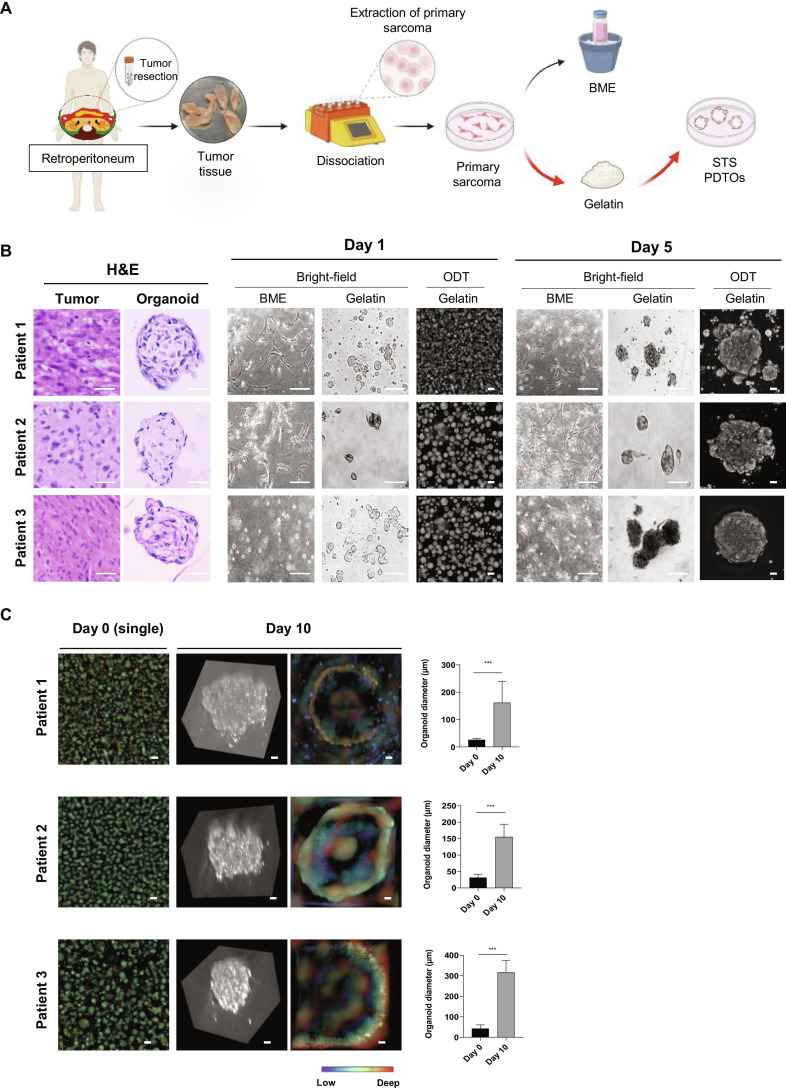
Generation of patient-derived tumor organoids (PDTOs) from retroperitoneal soft-tissue sarcoma (STS). (A) Schematic overview of the PDTO generation process from patient tumor samples using basement membrane extract (BME) and gelatin. (B) Representative images of sarcoma tissues and gelatin-based PDTOs (GbPDTOs) stained with hematoxylin and eosin (H&E; left). Scale bars: sarcoma tissue = 200 μm; organoid = 200 μm. Bright-field images of BME-based and GbPDTOs and corresponding optical diffraction tomography (ODT) images of GbPDTOs on days 1 (center) and 5 (right) of organoid formation. Scale bars: BME and gelatin = 200 μm; ODT = 25 μm. (C) Representative ODT depth images of GbPDTOs on days 0 and 10 and 3-dimensional (3D) depth-mapped views of day 10 organoids. Scale bar = 25 μm. The average organoid diameter for each patient is shown. ***P < 0.001 (Student *t* test).

The procedure for organoid generation is schematically illustrated in Fig. 1A. For PDTO establishment, patient-derived 2-dimensional (2D) cells were embedded at a final concentration of 5 × 10^5^ to 7 × 10^5^ cells in either BME (Bio-Techne R&D Systems, Minneapolis, MN, USA) or 20% gelatin (Samchun Chemicals, Seoul, South Korea) mixed with serum-free DMEM/F12 medium. The mixture was plated onto petri dishes (SPL Life Sciences Co., Ltd.). After the polymerization of BME or gelatin, a conditioned organoid culture medium was added. The organoid medium comprised advanced DMEM/F12 (Gibco) supplemented with 10% FBS (Thermo Fisher Scientific Inc.), 1% penicillin–streptomycin (Cytiva), 500 ng/ml recombinant human R-Spondin protein (Bio-Techne R&D Systems), 100 ng/ml human noggin (Gibco), 1× B27 supplement minus vitamin A (Gibco), 1 mM *n*-acetylcysteine (Sigma-Aldrich), 10 mM nicotinamide (Sigma-Aldrich), 50 ng/ml human epidermal growth factor (Novoprotein), 500 nM A83-01 (MedChemExpress, Monmouth Junction, NJ, USA), 10 μM SB202190 (Selleck Chemicals LLC, Houston, TX, USA), and 100 µg/ml Primocin (InvivoGen, San Diego, CA, USA). Organoid formation was monitored every 10 d. Gelatin-based PDTOs (GbPDTOs) were subsequently harvested for further analysis.

### Immunohistochemistry staining

Histopathological analysis was performed on the original tumor tissues and their corresponding PDTOs. Organoids were washed with phosphate-buffered saline and fixed in 4% paraformaldehyde (CellNest Inc., Korea) for 1 h at room temperature. Following fixation, samples were washed, transferred to 70% ethanol, paraffin-embedded, and sectioned at a thickness of 4 μm. Sections were stained with hematoxylin and eosin, deparaffinized, rehydrated, and incubated with primary antibodies (Table S1), followed by horseradish peroxidase (HRP)-conjugated secondary antibodies. Signal detection was performed using a diaminobenzidine substrate kit (Dako, a division of Agilent Technologies, Santa Clara, CA, USA), and nuclei were counterstained with hematoxylin (Vector Laboratories, Newark, USA).

### Seahorse assay

The oxygen consumption rates (OCRs) of 2D patient-derived sarcoma cells and PDTOs cultured in BME or gelatin were measured using the XF Cell Mito Stress Test kit (Agilent Technologies). Cells were seeded at 70% to 80% confluency in XFe24 Extracellular Flux Analyzer (Agilent Technologies) 96-well cell culture plates and incubated at 37 °C for 2 d. Before the assay, the growth medium was removed, and cells were rinsed with freshly prepared Seahorse XF assay medium (Seahorse Bioscience, a part of Agilent Technologies). A final volume of 200 μl of the assay medium was added to each well, and the plate was incubated for 45 to 60 min at 37 °C in a non-CO_2_ incubator. Mitochondrial OCR was then measured using the XF Cell Mito Stress Test kit (Agilent Technologies) on XF24 Extracellular Flux Analyzer. The OCR values were normalized to cellular DNA content.

### Label-free optical diffraction tomography

Optical diffraction tomography (ODT), a label-free quantitative phase imaging technique, reconstructs 3D images of organelles, cells, and organoids from multiple 2D holograms acquired at various angles [10]. ODT enables real-time, label-free imaging of live cells, providing quantitative data on refractive index (RI) and volume under different physiological conditions [11]. Detailed methodology for ODT setup and reconstruction is available in previous studies [10,12]. The ODT imaging and analysis were performed using a commercial ODT microscope equipped with a Mach–Zehnder interferometer (HT-X1, Tomocube Inc., Daejeon, Korea). Patient-derived 2D cells and organoids (cultured in both BME and gelatin) were imaged under light-emitting diode illumination (450-nm center wavelength) in a 5% CO_2_, 37 °C environment from day 0 to day 10. The 3D RI image reconstruction and quantitative analysis were performed using the TomoAnalysis software (Tomocube Inc.) [10]. While individual cells were challenging to distinguish in deeper PDTO regions, clear single-cell outlines were observable near the surface adjacent to the cover glass. Analyses focused on identifiable single cells from both 2D cultures and PDTO holograms. Small, uniformly circular intracellular structures with higher RI values, presumed to be lipid based, were quantified per cell.

### Confocal laser scanning microscopy

Patient-derived 2D cells and organoids cultured in BME and gelatin were chemically fixed with 4% paraformaldehyde, followed by immunofluorescence staining. Samples were then mounted onto glass slides for imaging using an inverted confocal laser scanning microscope (LSM880, Carl Zeiss AG, Oberkochen, Germany). For visualization, gelatin-cultured fixed cells and organoids were stained with 1 nM MitoTracker (Thermo Fisher Scientific Inc.); lipid droplets (LDs) were visualized using a LD dye (Biotium Inc., Fremont, CA, USA; dilution 1:1,000). Nuclei were counterstained with 4′,6-diamidino-2-phenylindole (DAPI) (Thermo Fisher Scientific) for 30 min at 37 °C. Fluorescence images were processed using the ZEN 3.1 software (Carl Zeiss AG) for visualization and quantitative analysis. Mitochondrial immunofluorescence was quantified and normalized to the DAPI signal using the ImageJ software (National Institutes of Health, USA) [13].

### Western blotting

For western blotting, 2D-cultured cells and organoids were digested with trypsin and washed with ice-cold phosphate-buffered saline. Cell lysis was performed in RIPA buffer (50 mM HEPES, pH 7.4; 150 mM NaCl; 1 mM EDTA; 2.5 mM EGTA; 1 mM DTT; and 1% Triton X-100), supplemented with a protease and phosphatase inhibitor cocktail (Sigma-Aldrich, St. Louis, MO, USA). Lysates were centrifuged at 13,000 rpm for 20 min to remove debris. Protein concentrations were quantified using the Bradford assay. Equal amounts of protein (10 μg) were loaded onto sodium dodecyl sulfate–polyacrylamide gel electrophoresis (gels (8%), separated electrophoretically, and transferred onto polyvinylidene fluoride membranes (Immobilon, Millipore Corporation, Billerica, MA, USA). Membranes were blocked in 5% nonfat dry milk diluted in Tris-buffered saline with 0.1% Tween-20 (20 mM Tris-HCl, pH 7.4; 150 mM NaCl; and 0.1% Tween-20) to minimize nonspecific binding. Primary antibodies against N-cadherin, AMP-activated protein kinase (AMPK), and actin were applied (Table S2). After 18 h of incubation, membranes were washed and treated with HRP-conjugated secondary antibodies (goat anti-rabbit). Signals were visualized using a chemiluminescence detection system (Amersham, Cytiva/GE Healthcare, Buckinghamshire, UK).

### Library sequencing and data analysis

Total RNA was isolated using TRIzol reagent (Invitrogen, Thermo Fisher Scientific Inc.) or Maxwell RSC miRNA Kits (Promega Corporation, Madison, WI, USA). RNA quality was assessed by Agilent 4200 TapeStation System (Agilent Technologies), and concentrations were quantified using Qubit Fluorometer (Thermo Fisher Scientific Inc.). Libraries for control and experimental samples were prepared using QuantSeq 3′ mRNA-Seq V2 Library Prep Kit FWD (Lexogen GmbH, Vienna, Austria) following the manufacturer’s protocol. Briefly, an oligo(dT) primer with an Illumina-compatible sequence was hybridized to total RNA, followed by reverse transcription. After RNA degradation, second-strand complementary DNA synthesis was initiated by a random primer containing an Illumina-compatible linker. The resulting double-stranded complementary DNA library was purified with magnetic beads, adapter-amplified, and further purified. High-throughput sequencing was performed as 75-bp single-end reads using the NextSeq 500/550 platform (Illumina Inc., San Diego, CA, USA). Data mining and graphic visualization were performed using the ExDEGA software (E-Biogen Inc., Seoul, Korea).

### Statistical analysis

Statistical analysis was performed using GraphPad Prism (Version 9.0, GraphPad Software, San Diego, CA, USA). Descriptive statistics and tests of significance were applied as appropriate. Unpaired or ratio-paired *t* tests were used for comparison between 2 groups, while a one-way analysis of variance was performed for those involving 3 or more groups. Statistical significance was set at a *P* value <0.05.

## Results

### Establishment of novel STS patient-derived organoids using gelatin

Cancer cells were isolated from the patient’s tissue before generating an organoid. Primary tumor cells were extracted from retroperitoneal sarcoma specimens (Table S1). These tumors are rare, accounting for <1% of all malignancies and 10% to 15% of STS cases. In this study, surgical specimens were obtained from the most prevalent retroperitoneal sarcomas—LPS and LMS [14]. Cultured sarcoma cells from all 3 patients displayed spindle-shaped, fibrotic morphologies (Fig. S1a).

Subsequently, STS PDTOs were generated from the extracted primary sarcoma cells of the 3 patients, patients 1 and 2 (LPS) and patient 3 (LMS), using gelatin. Moreover, to evaluate the suitability of gelatin as an ECM, GbPDTOs were compared with BME-based PDTOs (BbPDTOs), which are commonly used in organoid studies (Fig. 1A) [15]. After 5 d, BME failed to support 3D organoid formation. However, gelatin consistently enabled organoid-like 3D structures across all patient samples (Fig. 1B, day 5). Moreover, histological analysis further confirmed that GbPDTOs exhibited tissue-like morphology, resembling the corresponding tumors (Fig. 1B).

Organoids are complex 3D structures composed of diverse cell types within the ECM; therefore, conventional microscopy is limited in visualizing their architecture. Conversely, ODT enables real-time, high-resolution, label-free imaging by reconstructing 3D RI maps [16]. ODT imaging revealed that GbPDTOs exhibited proper formation and structural integrity of the organoids (Fig. 1B, day 5). Depth-resolved ODT images showed progressive increases in GbPDTO diameter from day 0 to day 10, indicating proper growth and maturation (Fig. 1C) [17]. Collectively, these results demonstrate that BME does not support the adequate formation of STS PDTOs, whereas gelatin effectively facilitates their generation.

### STS GbPDTOs retain the gene expression profiles of parental tumor tissues

**Fig. 2.**
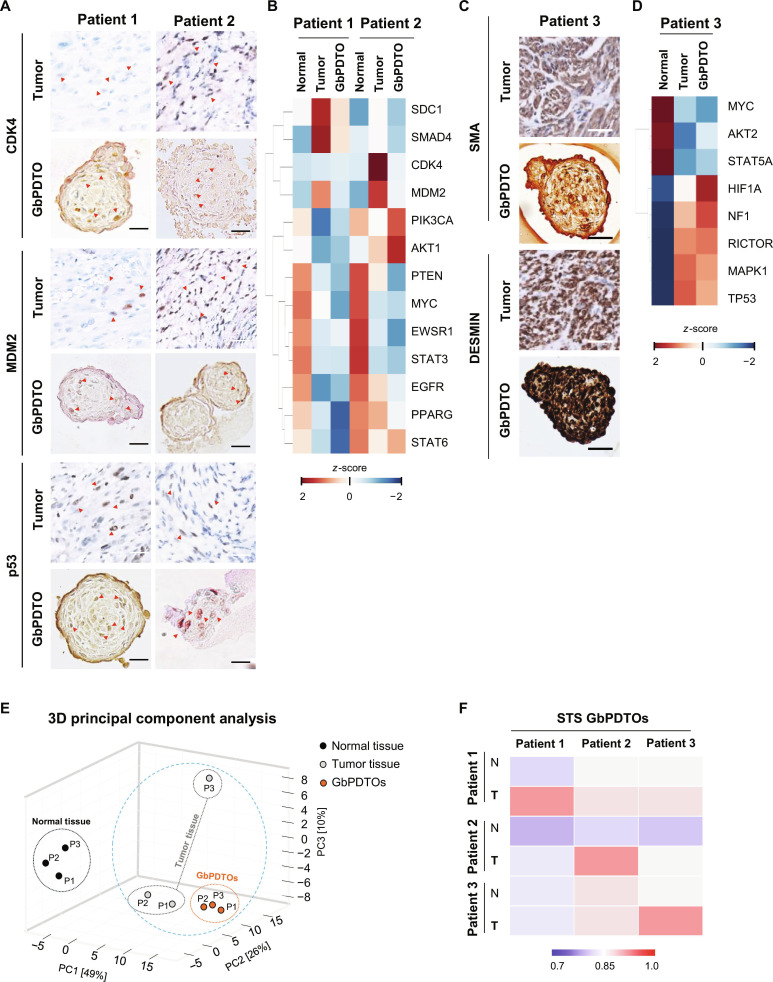
Characterization of GbPDTOs versus parental tumor tissues. (A) Immunohistochemistry (IHC) images of tumor tissues and GbPDTOs from patients with liposarcoma (patients 1 and 2) stained for cyclin-dependent kinase 4 (CDK4), murine double minute 2 (MDM2), and p53. Scale bars: tumor tissue = 50 μm; organoid = 200 μm. (B) Heatmap showing differential gene expression across normal tissues, tumor tissues, and GbPDTOs (patients 1 and 2). The color bar indicates *z*-scores (log_2_ fold changes): red = upregulated; blue = down-regulated. (C) IHC images of tumor tissues and GbPDTOs from a patient with leiomyosarcoma stained for smooth muscle actin (SMA) and DESMIN. Scale bars: tumor tissue = 50 μm; organoid = 200 μm. (D) Heatmap analysis of gene expression in normal, tumor, and GbPDTO tissues from patient 3. (E) 3D principal component analysis (PCA) plot illustrating the transcriptomic separation between normal tissues, tumor tissues, and GbPDTOs across 3 patients. (F) Pearson correlation matrix showing similarities in global gene expression among normal tissues, tumor tissues, and GbPDTOs. The color scale represents the Pearson correlation coefficient (*r*), with darker red indicating a stronger correlation.

Subsequently, GbPDTOs were evaluated for their expression of subtype-specific markers characteristic of each sarcoma type. Preserving the histological and molecular characteristics of the parental tumor is critical for the utility of PDTOs [4]. Immunohistochemistry (IHC) was performed to assess the expression of key diagnostic markers, including cyclin-dependent kinase 4 (CDK4), murine double minute 2 (MDM2), p53, smooth muscle actin (SMA), and DESMIN, in both GbPDTOs and their corresponding tumor tissues. LPS-derived organoids (patients 1 and 2) demonstrated positive expression of CDK4, MDM2, and p53 (Fig. 2A). Similarly, the organoid derived from patient 3 exhibited SMA and DESMIN expression, consistent with LMS characteristics (Fig. 2C).

Moreover, to determine whether PDTOs reflected the gene expression landscape of their tumors, RNA sequencing was performed on GbPDTOs, matched tumor tissues, and normal tissues from the 3 patients. Heatmap clustering of subtype-specific and oncogenic-pathway-related genes [18,19] revealed that PDTOs from patients 1 and 2 closely mirrored their tumor tissues, particularly in the expression of CDK4, MDM2, and myelocytomatosis oncogene (MYC). Conversely, normal tissues displayed distinct patterns (Fig. 2B). In patient 3, the GbPDTO maintained the tumor-like expression of oncogenes including MYC, serine/threonine kinase 2 (AKT2), and mitogen-activated protein kinase 1 (MAPK1), whereas tumor suppressors such as TP53 and neurofibromin 1 (NF1) were comparatively down-regulated (Fig. 2D). Principal component analysis revealed clear transcriptomic separation between normal tissues, tumor tissues, and GbPDTOs [20], with tumor organoids clustering closely with their parental tumors, indicating high transcriptomic fidelity. Although the LMS sample (sample 6) formed a distinct cluster from other sarcoma subtypes, its corresponding organoid remained adjacent to the original tumor cluster, supporting accurate representation (Fig. 2E). Pearson correlation analysis further confirmed strong transcriptomic concordance between GbPDTOs and their respective tumor tissues (Fig. 2F). Collectively, these findings demonstrate that GbPDTOs derived from all 3 patients robustly preserve the gene expression signatures of their parental sarcoma tissues, supporting their potential as representative tumor models.

### The transcriptomic profiling of GbPDTOs reflects the core oncogenic signatures of sarcoma

**Fig. 3.**
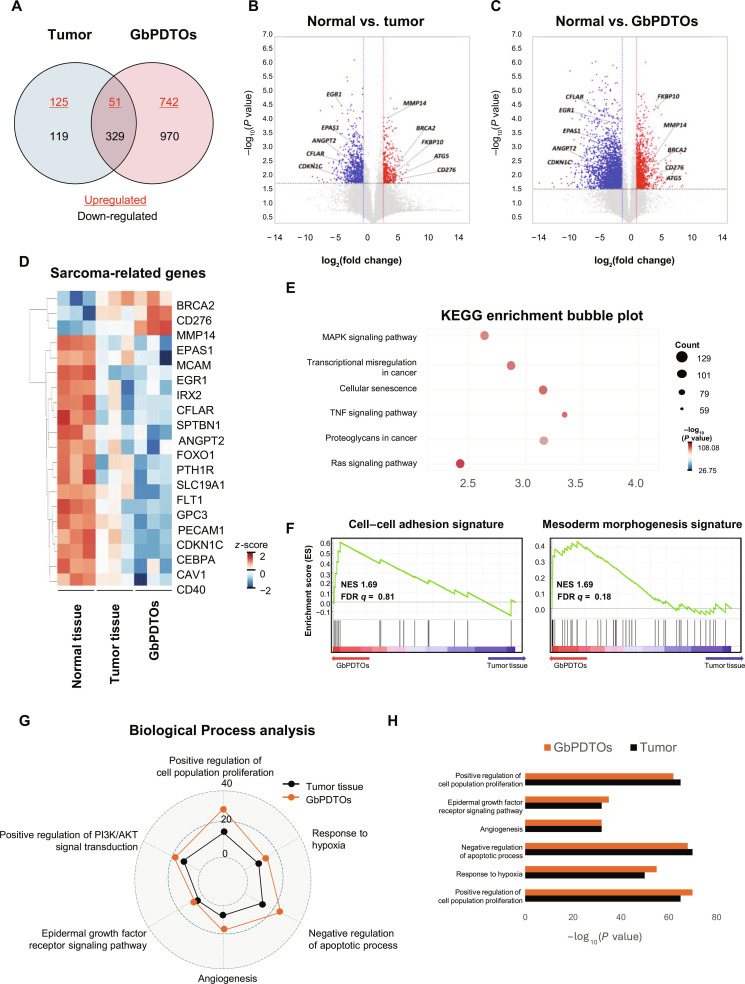
Transcriptomic and functional profiling of GbPDTOs versus original tumor tissues. (A) Venn diagram depicting the number of differentially expressed genes (DEGs) in tumor tissues and GbPDTOs relative to those in normal tissues. Upregulated and down-regulated genes are indicated in red and blue, respectively. (B and C) Volcano plots showing DEGs in normal vs. tumor tissues (B) and normal tissues vs. GbPDTOs (C). Red = upregulated; blue = down-regulated (cutoff: mean fold change > 2.0, *P* < 0.05). (D) Hierarchical clustering of selected sarcoma-related genes across normal tissues, tumor tissues, and GbPDTOs. The color bar shows *z*-scores (log_2_ fold change): red = upregulated; blue = down-regulated. (E) Bubble chart of Kyoto Encyclopedia of Genes and Genomes (KEGG) pathway enrichment analysis comparing GbPDTOs and tumor tissues. (F) Gene set enrichment analysis (GSEA) of cell–cell adhesion and mesoderm morphogenesis in GbPDTOs relative to those in tumor tissues. (G) Radar plots showing Biological Process (BP) enrichment analysis of 6 pathways in tumor tissues and GbPDTOs relative to those in normal tissues. (H) Comparison of biological processes enriched in tumor tissues and GbPDTOs. NES, normalized enrichment score; FDR, false discovery rate.

The gene expression profiles of GbPDTOs and sarcoma tissues were compared to evaluate how closely organoids reproduced the transcriptional landscape of primary tumors. Compared to the genes in normal tissues, 176 genes were upregulated, and 448 were down-regulated in tumor tissues, whereas GbPDTOs exhibited 793 upregulated and 1,299 down-regulated genes. Among them, 380 genes displayed concordant expression patterns (upregulated: 51 genes; down-regulated: 329 genes) in both tumors and GbPDTOs (Fig. 3A). Volcano plots highlighted these transcriptional differences and similarities between tumor tissues, GbPDTOs, and normal tissues (Fig. 3B and C). Moreover, hierarchical clustering of the top 20 genes commonly enriched in tumor tissues and GbPDTOs, selected from the GeneCards database relevance scores (Fig. 3D), revealed prominent upregulation of CD276, matrix metallopeptidase 14 (MMP14), endothelial PAS domain protein 1 (EPAS1), and forkhead box O1 (FOXO1), all of which are implicated in immune modulation, ECM remodeling, and hypoxia responses [21–23]. These genes have been associated with sarcoma progression and poor prognosis [22,24]. However, CD40, glypican 3 (GPC3), Iroquois homeobox 2 (IRX2), FOXO1, EPAS1, and cyclin-dependent kinase inhibitor 1C (CDKN1C) were consistently down-regulated in tumor tissues and GbPDTOs compared to those in normal tissues. The down-regulation of CD40 has been associated with poor prognosis in patients with STS [25], reflecting an immunosuppressive microenvironment that facilitates immune evasion [26]. These results suggest that low CD40 expression could serve as a negative prognostic marker and may inform immune-based therapeutic strategies for STS.

To assess the functional implications of differentially expressed genes (DEGs), Gene Ontology enrichment analysis was performed. Kyoto Encyclopedia of Genes and Genomes pathway enrichment analysis identified key oncogenic and tumor-progression-related signaling pathways associated with the DEGs, including “MAPK signaling”, “transcriptional misregulation in cancer”, “cellular senescence”, “TNF signaling pathway”, “proteoglycans in cancer”, and “Ras signaling pathway” (Fig. 3E) [27–29]. Furthermore, Biological Process enrichment analysis revealed important involvement of GbPDTO DEGs in cancer-related pathways, including “positive regulation of cell population proliferation”, “response to hypoxia”, “negative regulation of apoptotic process”, “angiogenesis”, “epidermal growth factor receptor signaling pathway”, and “positive regulation of PI3K/AKT signal transduction” (Fig. 3G). These pathways align with known cancer hallmarks, including enhanced motility, adaptation to hypoxia, transcriptional dysregulation, and sustained angiogenesis, all of which drive tumor progression (Fig. 3H) [30–32]. Gene set enrichment analysis revealed enrichment of mesoderm morphogenesis in GbPDTOs relative to tumor tissues (normalized enrichment score [NES] = 1.69, false discovery rate [FDR] *q* value = 0.18) (Fig. 3F). A trend toward enrichment of cell–cell adhesion pathways was also observed in GbPDTOs (NES = 1.69, FDR *q* value = 0.81). These results indicate that GbPDTOs preserve the gene expression profiles of primary sarcomas and maintain core oncogenic signaling pathways, supporting their validity as preclinical models.

### Mitochondrial homeostasis supports the generation of STS PDTOs

**Fig. 4.**
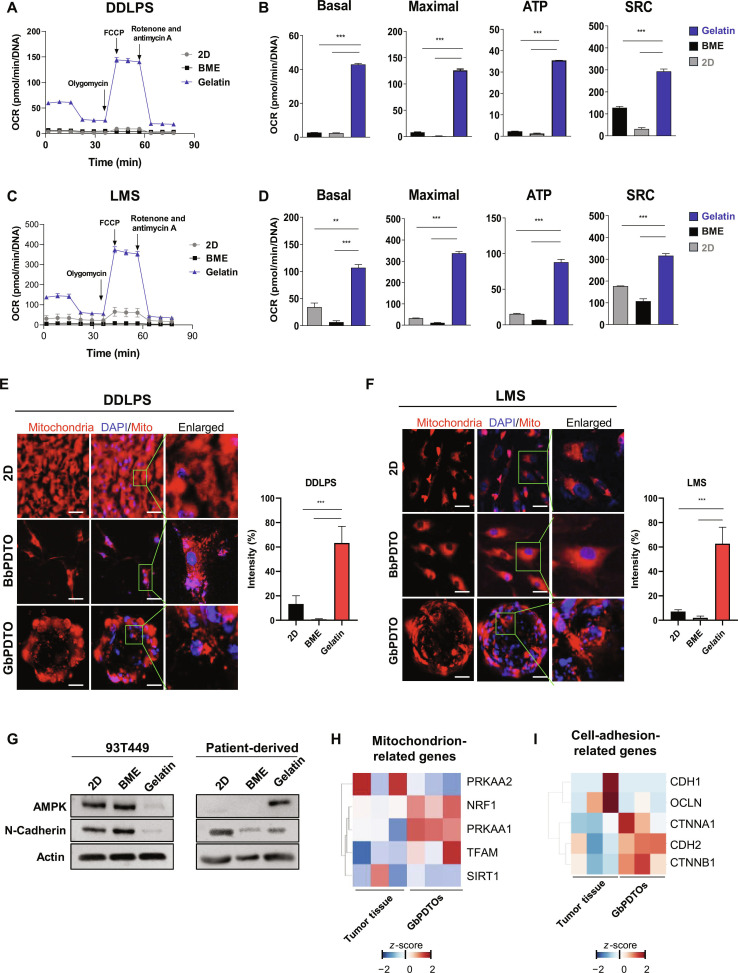
Gelatin modulates mitochondrial function and adhesion in STS organoids. (A and C) Time-dependent oxygen consumption rate (OCR) profiles of dedifferentiated liposarcoma (DDLPS) (A) and leiomyosarcoma (LMS) (C) cells cultured under 2-dimensional (2D), BME, and gelatin-based conditions. FCCP, carbonyl cyanide 4-(trifluoromethoxy) phenylhydrazone; Rot, rotenone; AA, antimycin A. OCRs were normalized to DNA concentration. (B and D) Quantification of basal respiration (Basal), maximal respiration (Maximal), ATP production (ATP), and spare respiratory capacity (SRC) derived from OCR. Data are presented as mean ± standard deviation (SD). **P < 0.01, and ***P < 0.001, one-way analysis of variance (ANOVA). (E and F) Confocal images of mitochondrial staining and fluorescence intensity normalized to 4′,6-diamidino-2-phenylindole (DAPI) in DDLPS (E) and LMS (F) cultured in 2D, BME, and gelatin-based conditions. Mitochondria: MitoTracker (red); nuclei: DAPI (blue). Scale bars = 200 μm. (G) Western blot analysis of N-cadherin and AMP-activated protein kinase (AMPK) in 93T449 liposarcoma and primary liposarcoma cells cultured under 2D, BME, and gelatin conditions. (H and I) Heatmap of mitochondrial and adhesion-related gene expression in tumor tissues and GbPDTOs. The color scale reflects the *z*-scores of log_2_-transformed values (red = upregulated; blue = down-regulated). BbPDTO, BME-based PDTO.

STS PDTOs were investigated to understand why they successfully formed in gelatin but not in commercially used BME. Gelatin has been reported to enhance oxidative metabolism by facilitating oxygen and nutrient diffusion, thereby improving energy homeostasis. It facilitates 3D cell–cell interactions and aggregation, processes essential for physiological functions [8]. Based on these properties, gelatin was hypothesized to improve mitochondrial function in primary sarcoma cells, enabling organoid formation. OCR analysis of LPS and LMS cells cultured in 2D, BME, or gelatin-based conditions (Fig. 4A and C) revealed significantly higher basal and maximal respiration, ATP production, and spare respiratory capacity under gelatin-based conditions compared to those of 2D and BME cultures (Fig. 4B and D). Immunofluorescence further showed that GbPDTOs exhibited a more distinct mitochondrial morphology and stronger fluorescence intensity compared to 2D cultures or BbPDTOs (Fig. 4E and F), consistently across both sarcoma subtypes. This result suggests that gelatin enhances mitochondrial activity and contributes to structural remodeling.

To validate these findings molecularly, the expressions of mitochondrial and adhesion-related proteins were examined. AMPK, a key regulator of energy metabolism, and N-cadherin, a key adhesion molecule critical for organoid formation, were both upregulated in GbPDTOs relative to those in BbPDTOs (Fig. 4G). Interestingly, in the 93T449 LPS cell line, BME-based organoids showed higher N-cadherin and AMPK expression than gelatin-based organoids; however, in patient-derived GbPDTOs, these markers were more strongly expressed under gelatin conditions. This contrast indicates that while BME may support organoid formation in established cell lines, gelatin is crucial for generating organoids from primary sarcoma cells.

Heatmap analysis of transcriptomic data confirmed the upregulation of mitochondrial biogenesis markers, including transcription factor A, mitochondrial (TFAM); sirtuin 1 (SIRT1); and nuclear respiratory factor 1 (NRF1), as well as mesenchymal adhesion genes, such as N-cadherin (CDH2) and catenin beta 1 (CTNNB1) in GbPDTOs (Fig. 4H). These findings highlight that gelatin modulates mitochondrial function and dynamics, increases AMPK and adhesion molecule expression, and facilitates organoid formation more effectively than BME. Collectively, these results support the use of gelatin as a suitable scaffold for generating STS PDTOs.

### GbPDTOs exhibit LD accumulation, reflecting a metabolic hallmark of sarcoma

LD accumulation is increasingly recognized as a hallmark of cancer, reflecting its role in tumor metabolism and progression [33]. In sarcoma, LDs contribute to tumor progression by enabling metabolic reprogramming and adaptation to hypoxic and inflammatory conditions. Their presence is often associated with high-grade tumors and may serve as a therapeutic target in lipid-metabolism-driven sarcomas, with growing evidence linking LDs to treatment resistance [34,35].

**Fig. 5.**
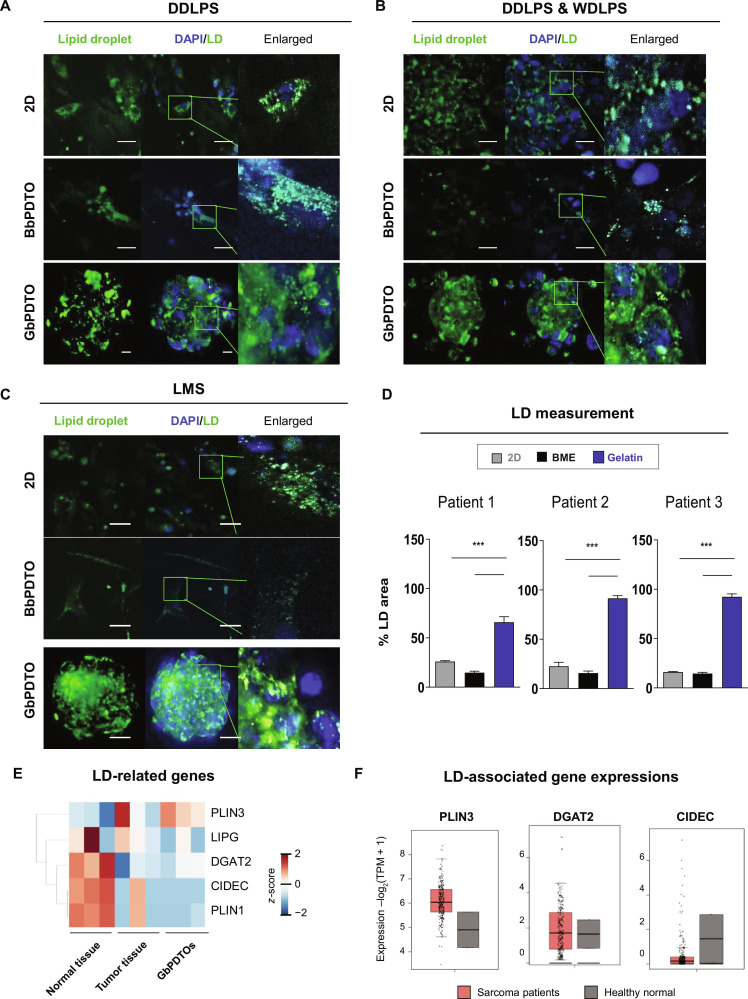
Gelatin enhances lipid droplet (LD) accumulation in patient-derived sarcoma organoids. (A to C) Representative confocal fluorescence images of LD accumulation (green) in patient 1, DDLPS (A); patient 2, DDLPS and WDLPS (B); and patient 3, LMS (C), cultured under 2D, BME, and gelatin conditions. Nuclei: DAPI (blue). Scale bars: 2D/BbPDTO = 200 μm; GbPDTO = 25 μm. (D) Quantification of the LD area in organoids from the 3 patients across 2D, BbPDTO, and GbPDTO cultures. Data are presented as mean ± SD. ***P < 0.001, one-way ANOVA. (E) Hierarchical clustering of LD-associated gene expression in normal tissues, tumor tissues, and GbPDTOs. The color bar reflects the *z*-scores of log_2_ fold changes (red = upregulated, blue = down-regulated). (F) Expression levels of perilipin 3 (PLIN3), diacylglycerol *O*-acyltransferase 2 (DGAT2), and cell-death-inducing DFFA-like effector (CIDEC) from the Gene Expression Profiling Interactive Analysis 2 (GEPIA2) database (normal = 2, sarcoma = 262). LIPG, lipase G, endothelial type.

To assess LD accumulation in GbPDTOs, 3D ODT imaging was used to quantify LD distribution across 3 different groups: 2D culture, BbPDTOs, and GbPDTOs. ODT enables label-free, fixation-free imaging under physiological conditions [36]. Results revealed that LD accumulation was significantly higher in GbPDTOs across all subtypes (Fig. 5A to C). For quantitative comparison, LDs were measured as the percentage of LD-positive area relative to the total imaging field. GbPDTOs exhibited a substantially greater LD-positive area compared to both patient-derived 2D cells and BbPDTOs (Fig. 5D).

Moreover, LD accumulation was evaluated in different sarcoma subtypes on day 0 and day 10 using label-free ODT imaging (Figs. S2 and S3). Distinct subtype-specific patterns emerged [37]. Consistent with this, our ODT-based imaging revealed that patients 2 and 3 (DDLPS with WDLPS, and LMS, respectively) exhibited higher LD accumulation at both time points than patient 1 (DDLPS only). The elevated LD levels in patient 2 may reflect the adipogenic characteristics of WDLPS [38]. Conversely, patient 1 (DDLPS) exhibited reduced LD accumulation, consistent with its diminished adipogenic potential. LMS may exhibit increased LD accumulation due to its higher oxidative metabolism and mitochondrial activity, typical of smooth-muscle-derived tumors [39]. These findings suggest that LD profiling may serve as a metabolic marker for STS subtype and microenvironmental adaptation. Notably, LD differences were evident across distinct sarcoma subtypes and LPS variants, indicating that LD phenotypes could influence sarcoma differentiation status.

Moreover, the expression profiles of LD-associated genes (perilipin 3 [PLIN3]; lipase G, endothelial type [LIPG]; diacylglycerol *O*-acyltransferase 2 [DGAT2]; and cell-death-inducing DFFA-like effector [CIDEC]) were analyzed. Heatmap analysis showed marked upregulation of PLIN3, whereas LIPG, DGAT2, CIDEC, and PLIN1 were down-regulated in tumor tissues and GbPDTOs compared to those in normal tissues (Fig. 5E). Validation using the public Gene Expression Profiling Interactive Analysis 2 sarcoma dataset confirmed consistent PLIN3 upregulation, and down-regulation of DGAT2 and CIDEC in tumor samples, mirroring our GbPDTO findings (Fig. 5F). These results support the translational relevance of GbPDTOs in replicating lipid metabolism and subtype-specific gene expression patterns of patient-derived sarcomas.

## Discussion

Unlike other prevalent carcinomas, STSs are rare and highly heterogeneous, accounting for <1% of all annual cancer diagnoses and comprising over 100 histological subtypes [1]. This rarity and diversity pose persistent challenges for both clinical trial design and disease modeling. PDTOs, which closely mimic their parental tumors, have emerged as valuable models for investigating tumor biology and developing targeted therapies in rare cancers, including STS [15]. Given their ability to mirror patient-specific treatment responses, PDTOs are increasingly recognized as key tools in precision oncology [40,41]. However, the lack of standardized protocols for generating STS PDTOs remains a significant barrier. Some subtypes of STS are indolent and challenging to culture as 2D tumor cells, while others are rare and understudied [41,42]. Most existing approaches rely on spheroid-based techniques, and only a few studies have explored them in depth [43]. For instance, successful organoid generation from angiosarcoma, a rare subtype of STS, was recently achieved only through fibronectin-coated dishes [44].

To overcome these limitations, an optimized gelatin-based method was developed for STS PDTO generation across 2 distinct STS subtypes. This approach improves on conventional organoid techniques by offering higher efficiency, reproducibility, and better representation of tumor heterogeneity. Furthermore, it provides a robust platform for biological investigation and therapeutic testing.

Initial attempts to establish STS organoids using conventional BME-based methods were unsuccessful. Elevated levels of MMPs in sarcoma cells degrade the BME matrix, hindering organoid formation and stability [6]. Therefore, gelatin was selected as an alternative ECM biomaterial, widely used for culturing mesenchymal stem cells, which share lineage similarities with the presumed cell of origin in STS [9]. Gelatin, commonly employed as a coating substrate, supports cell aggregation, a critical step in forming 3D structures [8,9]. Gelatin-based culture systems support enhanced cell proliferation and mitochondrial function. Reflecting these advantages, our method enabled the successful establishment of PDTOs from all 3 patient samples, yielding a 100% success rate whenever viable primary sarcoma cells were available. These findings highlight the reliability and reproducibility of gelatin-based organoid culture for STS.

Real-time ODT imaging further confirmed the development of well-organized 3D structures (Data File S1). ODT is a label-free quantitative imaging technique that reconstructs the 3D RI distributions of intracellular components. In particular, LDs, due to their high RI and uniform morphology, are more amenable to quantitative analysis via ODT compared to other organelles [45]. Unlike fluorescence-based imaging, ODT preserves sample integrity and allows noninvasive, high-resolution visualization of subcellular structures, including LDs. In this study, ODT enabled real-time monitoring of spatial density changes and morphogenetic transitions during organoid formation, providing insights into organoid compactness, core maturation, and cellular aggregation. Although resolution is limited in densely packed tissue regions, ODT effectively validated the structural integrity and organization of gelatin-based sarcoma organoids under physiologically relevant conditions.

Histological and transcriptomic analyses demonstrated a strong resemblance between GbPDTOs and their corresponding parental tumors. IHC showed the retention of key lineage markers, including DESMIN, SMA, and MDM2, reflecting subtype-specific characteristics within the organoids. Parallel transcriptomic profiling revealed strong concordance in the expression of cancer-related and subtype-associated genes, such as MDM2, CDK4, and TP53. Collectively, these findings highlight the molecular fidelity of GbPDTOs and support their value as preclinical sarcoma models.

LDs are increasingly recognized as indicators of cellular metabolic reprogramming, particularly in cancer-associated lipid remodeling [35]. In our study, 3D culture conditions considerably enhanced LD accumulation across all PDTOs compared to 2D monolayer cultures, with gelatin-based matrices producing the most significant effect, as shown in Fig. 5. Notably, the LD content varied significantly between patients, reflecting underlying differences in sarcoma subtypes. Organoids derived from patient 2 (DDLPS and WDLPS) exhibited substantially higher LD accumulation than those from patients 1 and 3, suggesting that tumor-subtype-specific metabolic programs may influence lipid metabolism. This finding aligns with previous reports indicating that LMS exhibits enhanced mitochondrial oxidative metabolism and lipid utilization compared to LPS [46]. These results suggest that gelatin provides structural support and improves mitochondrial function, both of which are essential for stable 3D growth. To the best of our knowledge, this is the first study to establish an STS PDTO model using a gelatin-based 3D culture platform.

Despite these promising outcomes, several limitations persist. First, the small sample size and limited subtype representation restrict the generalizability of this model. Validation across broader sarcoma subtypes with additional experimental data will be essential. Furthermore, expanding the IHC panel would facilitate more detailed histopathological characterization across sarcoma variants.

The current GbPDTO model lacks essential components of the native tumor microenvironment, including stromal, immune, and vascular compartments [47]. Future studies should incorporate co-culture systems or in vivo models such as patient-derived xenografts [48]. Moreover, organ-on-a-chip platforms may further refine tumor-microenvironment modeling, providing a promising complement to organoid systems [49,50]. Although the overall culture duration was limited, sarcoma organoids were stably maintained up to passage 5 for approximately 50 to 60 d, demonstrating acceptable stability within the constraints of primary tumor models (Fig. S1b). To address limitations in sample size and subtype diversity, a follow-up study is being conducted to expand the patient cohort and further validate reproducibility. Comprehensive mechanistic studies are also underway to examine the role of gelatin in regulating mitochondrial function, cell–cell adhesion, and organoid formation. In parallel, varying gelatin concentrations were tested to identify optimal culture conditions (Fig. S1c). Higher concentrations generated more compact organoids and upregulated AMPK and N-cadherin expression (Fig. S1d). These results suggest that gelatin matrices enhance mitochondrial function and cell–cell interactions critical for organoid formation.

**Fig. 6.**
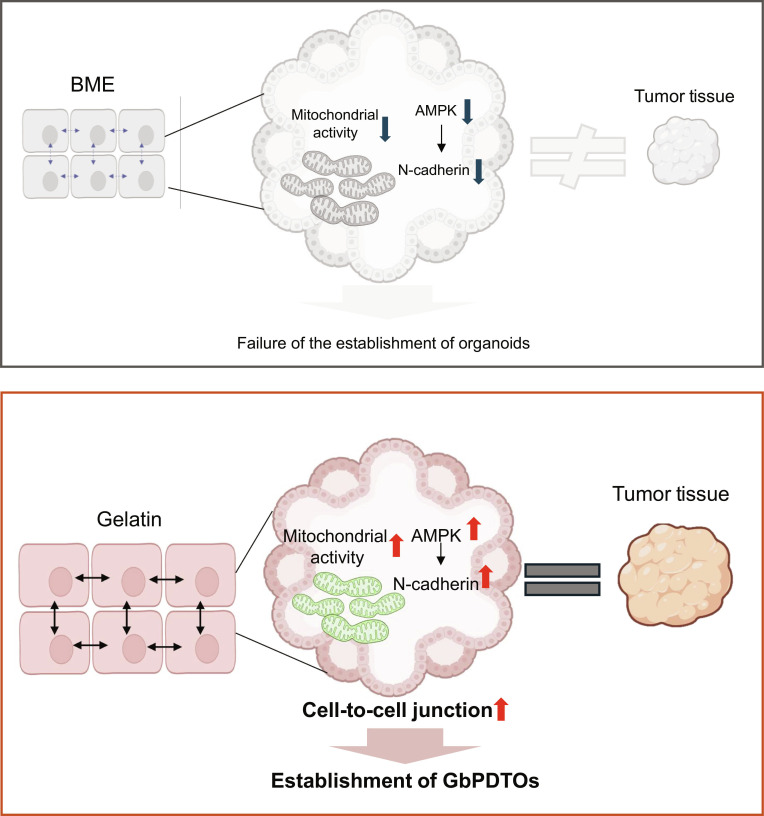
Illustration of PDTO generation from primary sarcoma cells in a gelatin matrix. Primary sarcoma cells embedded in a gelatin matrix exhibit enhanced mitochondrial activity, leading to AMPK pathway activation. Elevated AMPK expression induces N-cadherin upregulation and strengthens cell–cell junctions, contributing to the efficient generation of PDTOs compared to BME.

Establishing protocols for extended passaging and developing robust biobanking strategies will be essential for long-term investigations into sarcoma biology. In summary, this study introduces a novel, efficient, and reproducible gelatin-based culture system for generating STS PDTOs (Fig. 6). The platform faithfully reproduces the histological and molecular features of parental tumors while enabling analyses of tumor metabolic properties such as lipid remodeling. With further optimization and validation, this approach could be extended across diverse sarcoma subtypes, offering critical insights into sarcoma biology and potential therapeutic targets.

## Data Availability

The datasets generated and/or analyzed during the current study are available from the corresponding authors on reasonable request.
