# Supplementary material for: Gelatin-Based Soft-Tissue Sarcoma Organoids Recapitulate Patient Tumor Characteristics
Source: Biomater Res. 2025 Dec 9;29:0293. doi: 10.34133/bmr.0293 (PMC12686348; doi:10.34133/bmr.0293)
Supplement: Supplementary 1 — Tables S1 and S2 Figs. S1 to S3 Data File S1 [file bmr.0293.f1.zip › Supplementary Table 1.pdf]

Supplementary Table 1

Patient Number	Patient ID	Diagnose	Primary site	Age	Gender	Success of Primary Cell	Success of PDTO
Patient 1	AMC 003	[1]DDLPS	Retroperitoneum	39	M	Success	Success
Patient 2	AMC 013	DDLPS, [2]WDLPS	Retroperitoneum	50	M	Success	Success
Patient 3	AMC 007	[3]LMS	Retroperitoneum	80	F	Success	Success

[1]DDLPS: Dedifferentiated liposarcoma

[2]:WDLPS: Well-differentiated liposarcoma

[3]LMS: Leiomyosarcoma
