# Supplementary material for: Gelatin-Based Soft-Tissue Sarcoma Organoids Recapitulate Patient Tumor Characteristics
Source: Biomater Res. 2025 Dec 9;29:0293. doi: 10.34133/bmr.0293 (PMC12686348; doi:10.34133/bmr.0293)
Supplement: Supplementary 1 — Tables S1 and S2 Figs. S1 to S3 Data File S1 [file bmr.0293.f1.zip › Supplementary Table 2.pdf]

Supplementary Table 2

Antibody	Dilution	Product Code	Source
Pan-cytokeratin	IHC: 1:50	ab7753	Abcam
Vimentin	IHC: 1:500	MA511883	Invitrogen
P53	IHC 1:100	DO-1	Invitrogen
Ki-67	IHC: 1:100	SP6	Invitrogen
CDK4	IHC: 1:100	Ab108357	Abcam
MDM2	IHC: 1:100	IF2	Invitogen
S100	IHC: 1:100	MA512969	Invitrogen
Smooth muscle actin	IHC: 1:200	ab5694	Abcam
Desmin	IHC: 1:100	MA513259	Invitogen
NSE	IHC: 1:!00	PA527452	Invitogen
Nestin	IHC 1:1000	MA1110	Invitrogen
N-cadherin	Western Blot 1:1000	#4061	Cell Signaling
AMPK	Western Blot 1:1000	ab3759	Abcam
Actin	Western Blot 1:2000	A3854	Sigma
