# Supplementary figures and images for: Gelatin-Based Soft-Tissue Sarcoma Organoids Recapitulate Patient Tumor Characteristics
Source: Biomater Res. 2025 Dec 9;29:0293. doi: 10.34133/bmr.0293 (PMC12686348; doi:10.34133/bmr.0293)

# Supplementary Figure 1

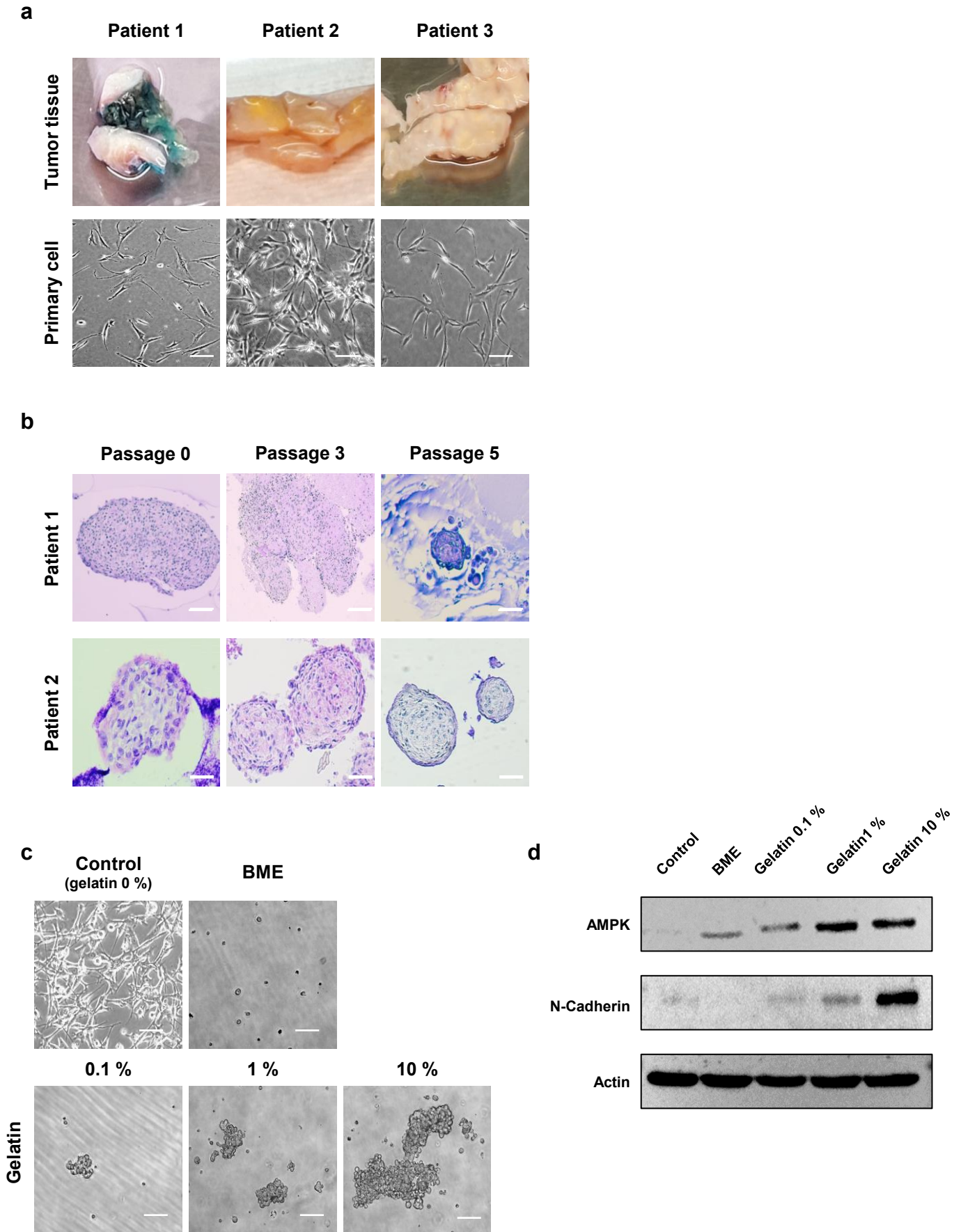

Supplement: Supplementary 1 — Tables S1 and S2 Figs. S1 to S3 Data File S1 [file bmr.0293.f1.zip › Revised figures_Supplementary figure 1.pdf]

a

Day 0

GbPDTOs

Enlarged

Patient 1

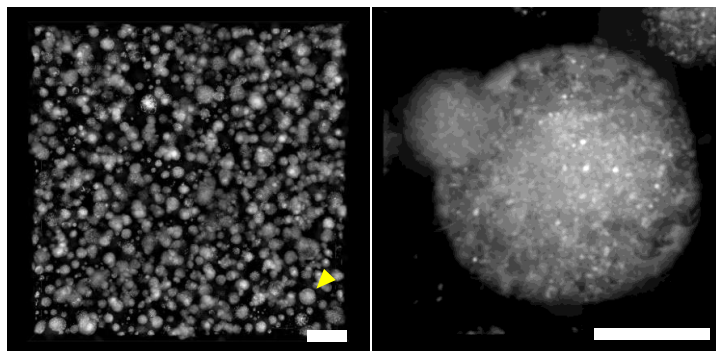

Patient 2

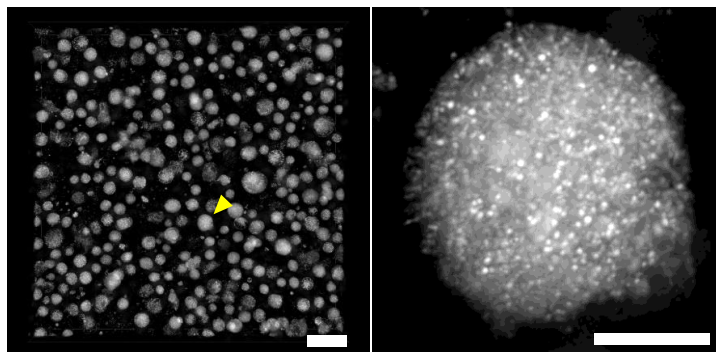

Patient 3

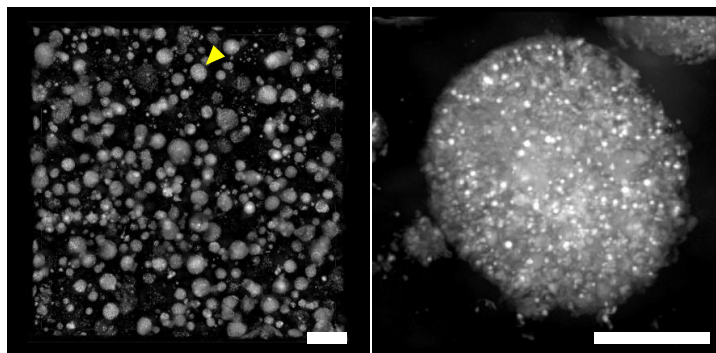

b

Day 0

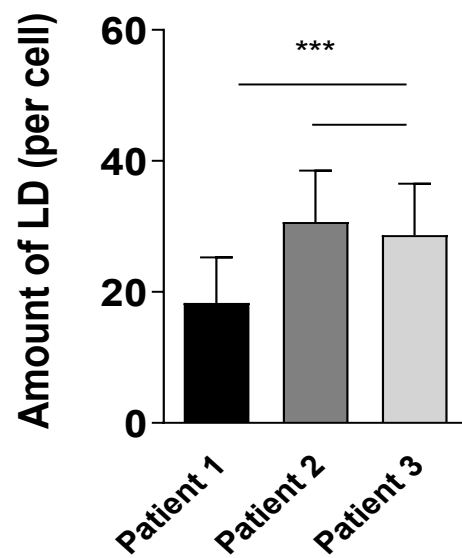

Supplement: Supplementary 1 — Tables S1 and S2 Figs. S1 to S3 Data File S1 [file bmr.0293.f1.zip › Revised figures_Supplementary figure 2.pdf]

a

Day 10

Supplementary Figure 3

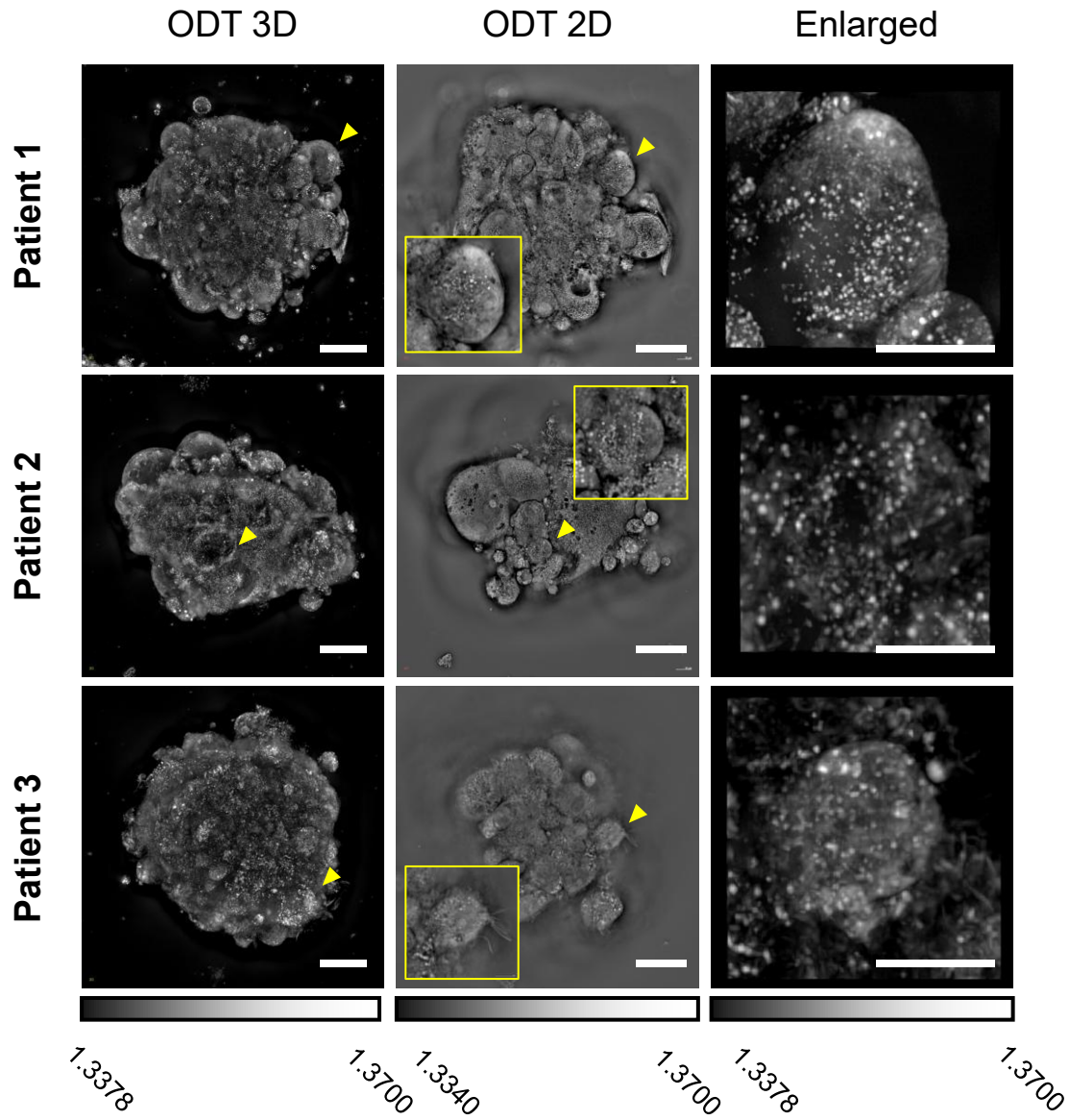

b

Day 10

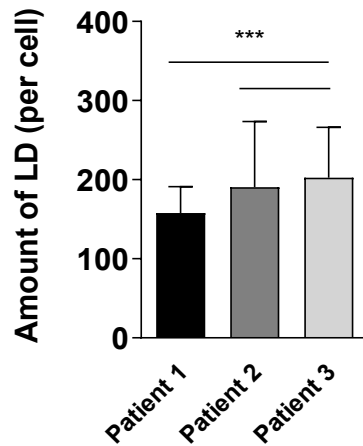

Supplement: Supplementary 1 — Tables S1 and S2 Figs. S1 to S3 Data File S1 [file bmr.0293.f1.zip › Revised figures_Supplementary figure 3.pdf]
